# Global database of matched *Plasmodium falciparum* and *P. vivax* incidence and prevalence records from 1985–2013

**DOI:** 10.1038/sdata.2015.12

**Published:** 2015-08-18

**Authors:** Katherine E. Battle, Carlos A. Guerra, Nick Golding, Kirsten A. Duda, Ewan Cameron, Rosalind E. Howes, Iqbal R.F. Elyazar, J. Kevin Baird, Robert C. Reiner, Peter W. Gething, David L. Smith, Simon I. Hay

**Affiliations:** 1 Spatial Ecology and Epidemiology Group, Tinbergen Building, Department of Zoology, University of Oxford, South Parks Road, Oxford OX1 3PS, UK; 2 Sanaria Institute for Global Health and Tropical Medicine, Rockville, Maryland 20850, USA; 3 Eijkman-Oxford Clinical Research Unit, Jalan Diponegoro No 69, Jakarta 10430, Indonesia; 4 Nuffield Department of Medicine, Centre for Tropical Medicine, University of Oxford, Oxford OX3 7FZ, UK; 5 Indiana University School of Public Health, Bloomington, Indiana 47405, USA; 6 Fogarty International Center, National Institutes of Health, Bethesda, Maryland 20892, USA

**Keywords:** Epidemiology, Malaria, Literature mining, Data mining

## Abstract

Measures of clinical incidence are necessary to help estimate the burden of a disease. Incidence is a metric not commonly measured in malariology because the longitudinal surveys required are costly and labour intensive. This database is an effort to collate published incidence records obtained using active case detection for *Plasmodium falciparum* and *Plasmodium vivax* malaria. The literature search methods, data abstraction procedures and data processing procedures are described here. A total of 1,680 spatio-temporally unique incidence records were collected for the database: 1,187 for *P. falciparum* and 493 for *P. vivax*. These data were gathered to model the relationship between clinical incidence and prevalence of infection and can be used for a variety of modelling exercises including the assessment of change in disease burden in relation to age and control interventions. The subset of data that have been used for such modelling exercises are described and identified.

## Background & Summary

The global clinical burden of malaria has proven difficult to enumerate. Previous efforts have estimated the clinical incidence of *Plasmodium falciparum* malaria using adjusted case reports^1,2^, modelling based on study-level incidence and mortality rates^3^ and cartographic modelling techniques^4–6^. All of these methods require malaria incidence measures in some part of their estimation or validation procedures. The surveillance-based approach relies on routinely reported case numbers, which are adjusted by country to account for incomplete reporting, the proportion of cases confirmed with routine diagnostics, and health facility use and access^1^. The Global Burden of Disease (GBD) study uses an amalgam of three methods to estimate case incidence. First, reported cases are used from a small subset of countries deemed to have reliable reporting. Second, corrected reporting (similar to the surveillance-based approach methods) is applied to a larger set of countries. And third, study-level incidence, such as the records reported here, along with mortality rates and a variety of parameters such as age and detection methods are used to model country-level incidence^3^. The cartographic approach, employed by the Malaria Atlas Project (MAP), also uses a tiered approach. Case estimates from countries with reliable estimate are used directly. Regions designated as unstable transmission regions^6,7^ are assigned an incidence of 0.1 cases per 1,000 per year. For regions without accurate reporting in areas stable malaria transmission, a modelled relationship between study-level measures of incidence matched in space and time to prevalence surveys is applied to a smooth endemicity (prevalence) surface^7^ and multiplied by a global population grid^4^. Such matched incidence and prevalence data are presented here.

Whilst approximations of ‘non-*P. falciparum*’ malaria exist, the burden of *Plasmodium vivax* malaria is considered to be largely unknown^8–10^. The primary reason for these knowledge gaps is that measures of malaria incidence are rarely undertaken as they are logistically demanding and thus expensive. To accurately measure clinical incidence of malaria, longitudinal studies must be conducted that include regular visits made to communities to check for symptomatic individuals through active case detection (ACD)^11^. The database described here aimed to compile as many ACD studies for *P. falciparum* and *P. vivax* as possible from 1985 to 2013 and represents a significant expansion upon previously published assemblies of incidence data^5^.

A more commonly measured malaria metric is prevalence, also known as parasite rate (PR)^12^. As demonstrated by Patil *et al.*^5^, Cameron *et al.*^13^ and Battle *et al.*^14^, ACD incidence records can be matched to PR measures to model the relationship between prevalence of infection and incidence of clinical disease. These models then have the potential to transform existing endemicity maps^7,15^ into global burden estimates with known precision^5^. For this purpose, each ACD observation in this database has been matched to a concurrently measured PR value or an extracted spatially and temporally matched modelled PR^7,15^.

All data curation and abstraction procedures to obtain the 1,680 incidence records, including geo-positioning and prevalence matching, are described here. The structure of the final database and technical validation efforts are also described along with notes to facilitate the replication of the analyses in Cameron *et al.*^13^ and Battle *et al.*^14^ Such validation yields a powerful mathematical tool supporting efforts to reliably estimate global burdens of disease imposed by the parasites causing human malaria.

## Methods

### Data collection

Here we provide additional detail on methodology to that provided in Cameron *et al.*^13^ and Battle *et al.*^14^, which utilize only a subset of the data presented here. PubMed (http://www.ncbi.nlm.nih.gov/pubmed) was searched on 27 November 2013 using the following search string: ((malaria[MeSH Terms]) AND (‘Incidence’ [Mesh] OR ‘Epidemiology’ [Mesh] OR ‘epidemiology’ [Subheading])) AND (‘1985/01/01’[Date—Publication]: ‘3000’[Date—Publication]). This selected references published after 1 January 1985 and the Medical Subject Headings (MeSH; http://www.ncbi.nlm.nih.gov/mesh) ensured that all pseudonyms were included in the search. The cut off of 1985 was used to match the year range of PR records included in the MAP database^16^. The literature search returned 11,272 citations and was augmented with a further 25 references from previously published analyses^5,17^. A total of 15 search strings were tested varying the terms used and application of MeSH terms. The number of references returned ranged from 58 (((malaria[MeSH Terms]) AND ‘active case detection’) AND (‘1985/01/01’[Date—Publication]: ‘3000’[Date—Publication])) to 1,291,787 (((((malaria[MeSH Terms]) AND incidence[MeSH Terms]) OR epidemiology[MeSH Terms]) OR epidemiology[MeSH Subheading]) AND (‘1985/01/01’[Date—Publication]: ‘3000’[Date—Publication])). The search string used was chosen because it was a reasonable number of titles for a small team to sort through, while still capturing the majority (87%, 55/63) of the references used in a previously published collection^5^.

Abstracts were reviewed to determine if the reference might contain *P. falciparum* or *P. vivax* incidence data. References excluded at this stage included reviews, case studies, vector-only, reports on animal or non-*P. falciparum* or *P. vivax* malaria, reports of imported malaria and technical articles including mathematical modelling and genetic analyses. The full list of 11,297 references was narrowed down to 898 references for full text review. Seventy-eight references known to contain incidence data from the work by Patil *et al.*^5^ and Griffin *et al.*^17^ were also set aside.

**Figure 1:**
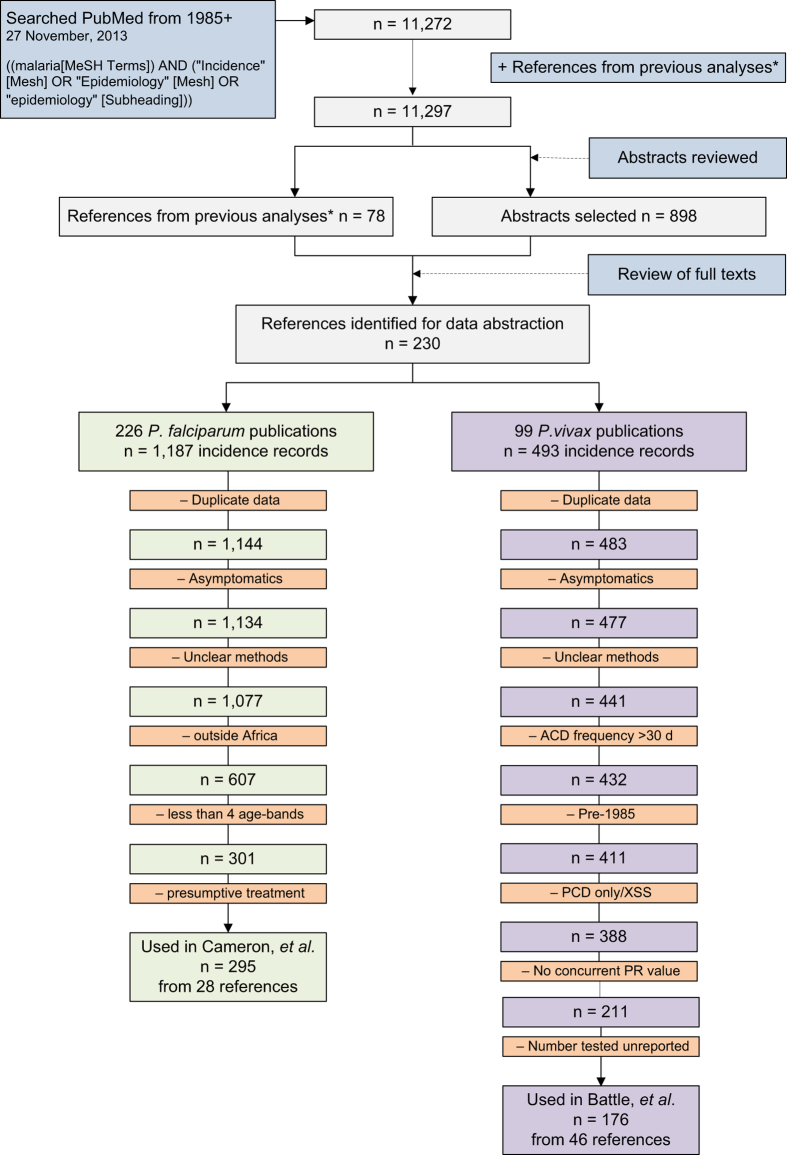
Schematic overview of the literature search procedure and results. The data exclusions to obtain clinical incidence records of use for model implementation for the *P. falciparum* (Cameron *et al.*^13^) and *P. vivax* (Battle *et al.*^14^) models are also shown. References from previous analyses* include those used by Patil *et al.*^5^ and Griffin *et al.*^17^

Full texts were obtained for the 976 references identified for review. The criteria for inclusion were: (i) longitudinal studies using ACD, (ii) symptomatic/clinical cases were the subjects of detection, (iii) studies were conducted in the general community (not patient sub-groups or hospital-based studies), and (iv) diagnosis using microscopy or rapid diagnostic test (RDT). Studies done only on pregnant women were excluded due to their increased susceptibility to malaria^18^. Conversely, studies using only infants aged less than three months were excluded due to their potential temporary immunity to *P. falciparum* from maternal antibodies^19^. *Plasmodium vivax* has been shown to cause significant morbidity in young infants^20,21^, however none of the studies conducted on infants measured *P. vivax* incidence. In-house language skills only allowed for the inclusion of articles written in English, French, Portuguese and Spanish. Twelve publications out of the 976 identified for review were in other languages (one Turkish and 11 Chinese) and therefore excluded. Articles that did not have enough information to determine the number of cases and the person-time observed (length of follow up for each cohort member) were excluded. Initially, there were no restrictions placed on the length or frequency of follow up, as long as it was explicitly reported. Based on the above criteria, data were abstracted from a total of 230 references, the majority of which measured incidence of *P. falciparum* malaria. Data on *P. falciparum* and *P. vivax* incidence were identified in 226 and 99 of these references, respectively. Literature review procedures are outlined in Fig. 1.

### Geo-positioning

**Figure 2:**
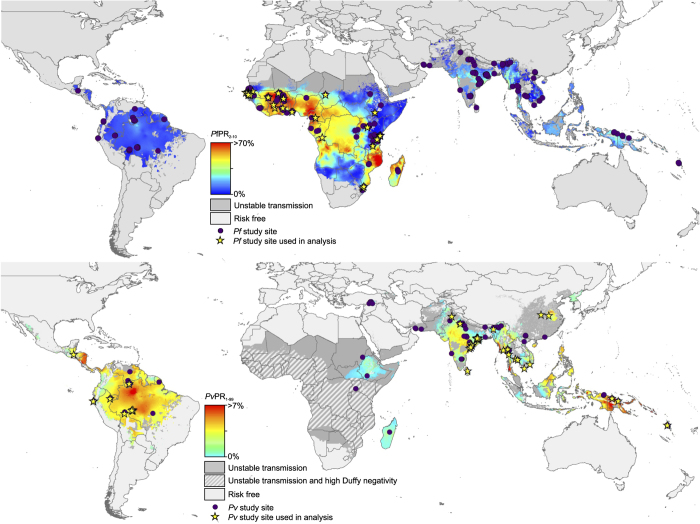
Distribution of incidence records of 2010 prevalence surfaces for *P. falciparum* and *P. vivax*. The geographic locations of the incidence records for *P. falciparum* (top panel) and *P. vivax* (bottom panel) are shown over the model-based geostatistics (MBG) point estimates of the annual mean *Pf*PR_2-10_ and *Pv*PR_1-99_ for 2010 within the spatial limits of stable limits of transmission (annual parasite index (API) ≥0.1 per 1,000 *per annum* (p.a.)), displayed on a continuum from blue (0% PR) to red (70% PR for *P. falciparum* and >7% PR for *P. vivax*). Dark grey areas were predicted to be unstable (API ≤0.1 per 1,000 p.a.) and light grey areas were classified as risk free. Areas in which Duffy negative allele frequency is predicted to exceed 90% (ref. 29) are shown in hatching for additional context in the *P. vivax* map. Study sites used in the *P. falciparum* (Cameron *et al.*^13^) and *P. vivax* (Battle *et al.*^14^) models are shown as yellow stars and other sites included in this dataset not used in the cited analyses are shown as purple points.

**Figure 3:**
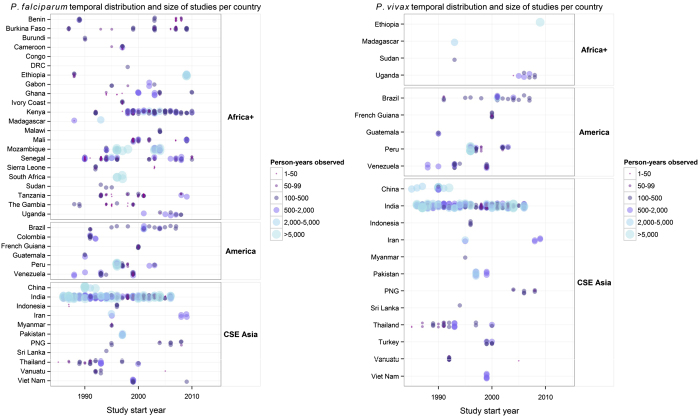
Temporal distribution and person-years observed for all incidence records by region and country. *Plasmodium falciparum* (left panel) and *P. vivax* (right panel) records are shown as points along an axis of the study start year. The points vary in colour and size based on the number of person-years observed in each study, such that studies with smaller sample sizes are small dark purple points and larger studies are large light blue points. The points are jittered so that overlapping points can be seen.

**Figure 4:**
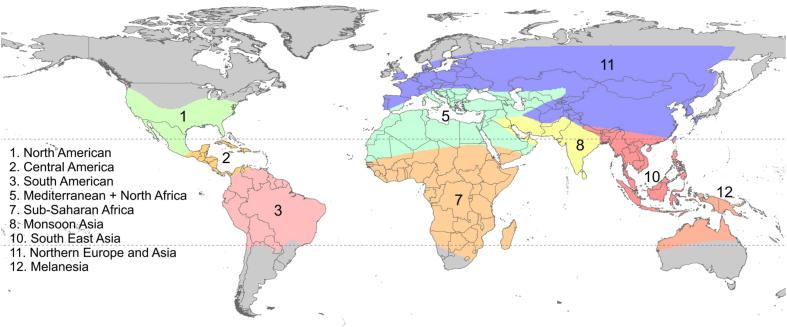
Geographic zones of varying malaria epidemiology and *P. vivax* relapse phenotypes. The zones were used by Battle *et al.*^27^ to illustrate large-scale patterns in of relapse behaviour. Zones 4, 6 and 9 are not shown because they were joined with other zones as described in Battle *et al.*^27^

All available location information was extracted from the published sources. Incidence records were first positioned to a MAP region: Africa+ (Africa plus Saudi Arabia and Yemen), the Americas, and Central and Southeast Asia (CSE Asia). The number of records by species and region are shown in Table 1. Next, they were assigned to a country (based on 2013 boundaries) and place. The place was considered the location of the study and latitude and longitude coordinates for each site were found using values given in the paper (converting to decimal degrees where necessary) or where this was unavailable, manually digitized using Google Maps^22^ or Microsoft Encarta^23^. Contextual information from the paper was used to differentiate when two places with the same name were within one country or to narrow down a region to be scanned for names that could be different spellings or translations of the site name (for example, Sissé rather than Cisse). If the site was a village, town or city, the latitude and longitude were taken from centre, unless a specific part of the town or city were specified as the study site. If the only location information given was a larger area such as a district administrative unit, as defined by the Food and Agriculture Organization (FAO) Global Administrative Unit Layers (GAUL) coding^24^, the centroid of the region was found using geographic information systems (GIS) software^25^. If the location could not be determined by any of these means, the authors were contacted for further information. The locations of each record are shown in Fig. 2 and the distribution of the data records over time are shown by country in Fig. 3. Records were also matched to zoo-epidemiological zones originally defined by Macdonald^26^ and modified by Battle *et al.*^27^ to describe the geographic variation observed in *P. vivax* relapse phenotypes (Fig. 4). These classifications enable the relationship between prevalence and incidence to be modelled separately by region.

### Calculating incidence

Incidence describes the number of events that occur within a specified time period. In this context, the events are symptomatic cases (fever cases confirmed by RDT or microscopy) of *P. falciparum* or *P. vivax* identified within a study population. Cases of *P. vivax* may arise from new mosquito-borne infections, recrudescence from treatment failure or relapses from hypnozoites (the dormant liver stage). Because there is no reliable way to differentiate relapses from new infections or recrudescence, the incidence of *P. vivax* reported here includes the cases from all origins.

Values for the number of cases and time period the study population was observed (recorded in person-years) were needed for modelling purposes and every effort was therefore made to extract or derive those values. If incidence was given, then the number of cases or person-time was derived from other information provided in the publication. In the few studies that provided age-specific data without age-specific population data, a general population structure was applied^28^ to the whole population to determine the size of the composite age groups. Where person-time was not explicitly reported, this was calculated by multiplying the population number by the length of the study period. This was necessary for the majority of studies for both *P. falciparum* (61%, 722/1187) and *P. vivax* (77%, 378/493).

### Matching prevalence to incidence

**Figure 5:**
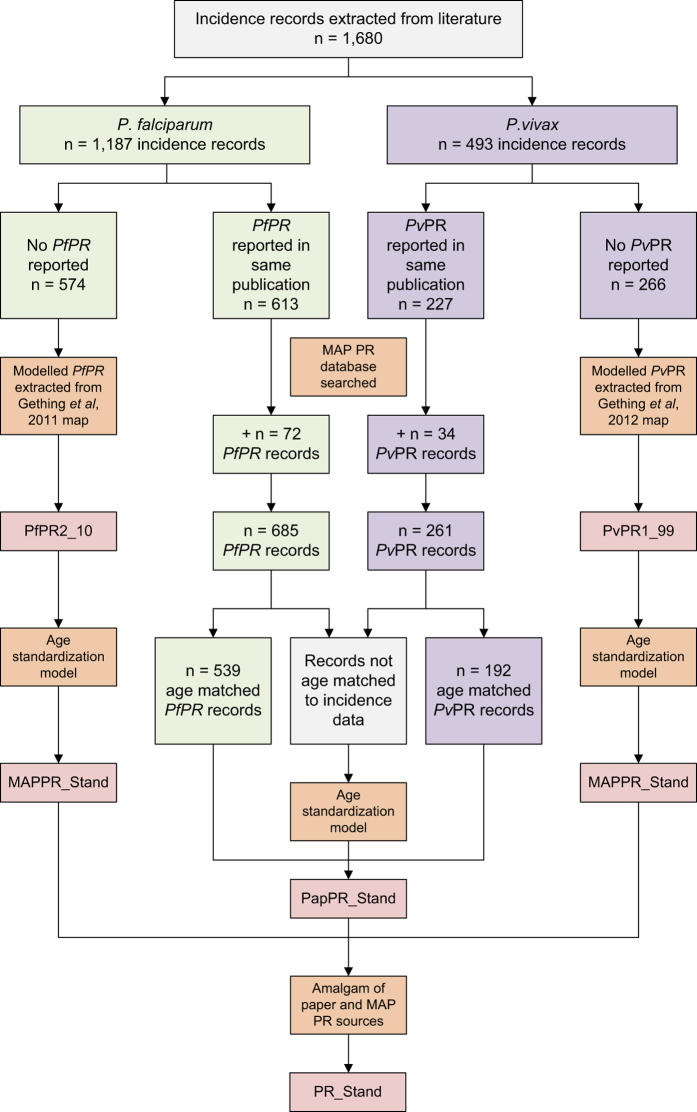
Schematic overview of the procedure of matching prevalence to incidence. Rectangles referring to all data are shown in grey, *P. falciparum* data in green, and *P. vivax* data in purple. Orange rectangles indicate data processing procedures and red rectangles symbolize fields in the final database.

So that the data may be used to model the relationship between prevalence (PR) and incidence of clinical disease, an estimate of prevalence was spatially and temporally matched to each incidence record in the database. If the incidence publication source contained empirical prevalence data, this was abstracted to provide a space-time match between PR and incidence. Averages were taken for those studies that reported more than one cross-sectional survey (XSS) for the same community. The number and timing of each survey was recorded where available. Half of the records had PR data available from the same reference (840/1680). Some additional space-time prevalence matches were added using the MAP PR database^16^ if a separate publication measured PR in the same community and year as the incidence data (6%, 97/1680). For the remaining 44% of the data (743/1680) without concurrent prevalence data, PR values were extracted for all incidence records using 2010 point estimates of the annual mean modelled *P. falciparum*^7^ and *P. vivax*^15^ endemicity values (shown in Fig. 1) using GIS software^25^. For *P. vivax*, areas where Duffy negative allele frequency was predicted to exceed 90% (ref. 29) is shown in hatching and *P. vivax* PR was predicted at <1% in those areas. The PR values for *P. falciparum* were predicted for two to ten year-olds, whereas for *P. vivax*, the predictions were made for all ages (one to 99 years). Because these age ranges did not always correspond to age groups in the incidence data, these data were age standardized using a bespoke model parameterized for *P. falciparum*^30^ and *P. vivax*^15^. The age standardization model was also applied as needed to PR values obtained from publications, as not all prevalence records had matching age ranges to the incidence data. The number of parasite positive individuals was then adjusted to match the age-standardized prevalence value. See results in Data Citation 1. A schematic illustration the process of matching prevalence and incidence data is shown in Fig. 5.

### Code availability

All age standardisation routines were implemented in an open-source software package ageStand^31^, implemented in the R statistical programming environment^32^. The package contains one function, convertPrevalence, which simplifies the conversion of prevalence estimates between age bounds. Five arguments were specified for the function. The first, prevalence, is a vector specifying the prevalence, or which PR field in this case, to convert from. Next, age_min_in and age_max_in, are vectors that specify the minimum and maximum ages associated with the estimates given in prevalence.
age_min_out and age_max_out are vectors that provide the lower and upper bounds of the age range that the prevalence is to be converted to. Finally, the parameters argument specifies a set of parameters to be used in the model and was set to ‘Pf_Smith2007’ for all *P. falciparum* conversions and ‘Pv_Gething2012’ for all *P. vivax* conversions, referring the papers where the models were originally published for each species^30,33^.

## Data Records

Data on each species was abstracted separately and data were disaggregated by age groups where possible. Values were input into a spreadsheet containing 63 fields:

### 1. Identification

ENL_ID. Data source identification number.

PI_ID. Unique identifier for each record.

INC_AUTHOR. First author surname of incidence data publication.

INC_PUBYEAR. Publication year of incidence data publication.

GRIFFIN. Identifies a record used in the analysis by Griffin *et al.*^17^

CAMERON. Identifies a record used in the analysis by Cameron *et al.*^13^

BATTLE. Identifies a record used in the analysis by Battle *et al.*^14^

EXCLUSION. Potential exclusion criteria as described in Table 2.

SPECIES. *P. falciparum* (Pf) or *P. vivax* (Pv).

### 2. Geo-positioning

REGION. MAP regions are America, Africa+ and CSE Asia.

COUNTRY. Country where ACD was conducted.

ACD_LOCATION. The town/village/district where the ACD was conducted.

LAT. Latitude in decimal degrees (WGS1984 datum).

LONG. Longitude in decimal degrees (WGS1984 datum).

LATLONG_SOURCE. Source of the coordinates: Paper (from the publication), Google^22^, Encarta^23^, Other (other online sources or databases), Pers. Comm. (personal communication, usually reporting GPS-read coordinate), GIS (centroid of administrative unit found using ArcGIS)^25^, or Combination (a combination of the aforementioned methods).

GEOPOS_NOTES. Further information about how geo-positioning was carried out.

EPIZONE. Numerical code for geographic epidemiological zones as defined in Battle *et al.*^27^ and as shown in Fig. 4.

BATTLEZONE. The full name of the EPIZONE described above.

### 3. Incidence time

START_MONTH. Survey starting month.

START_YEAR. Survey starting year.

END_MONTH. Survey ending month.

END_YEAR. Survey ending year.

TIME. Number of years of observation, using fractions to represent <1 year (1 for 12 months, 0.67 for 8 months, 1.33 for 16 months, etc.)

TIME_CAT. Categorizes the TIME column into six categories for the purpose of further exclusion if needed (<6 months, 6–11 months, 12 months, 13–23 months, 24 months, >24 months).

FREQ_ACD. Frequency of ACD written as text (every 2nd day, weekly, fortnightly, etc.). Weekly* indicates a record where the frequency of ACD was not explicitly reported in the study and assumed to be one week.

FREQ_ACD_NUM. This will express the frequency of ACD numerically should scaling be applied downstream. Records the number of days between each visit (every 2nd day=2, every fortnight=14, every month=30).

PCD. Passive Case Detection. *Yes/No* for if Passive Case Detection was conducted alongside the ACD.

POP. Number of people observed for TIME; the study population.

d. The number of positive species-specific clinical cases. Asymptomatic and mixed infections were not included. Mixed infections were often a negligible proportion of the total infections and present a challenge because it is not possible to determine the parasite that caused the symptomatic episode.

PYO. If person-time is specifically reported in the paper, that value was used after converting to person-years. If person-time was not explicit, the length of the study period (TIME) was multiplied by the study population (POP) such that PYO=TIME * POP.

PYO_APPR. PYO approximated. This is a binary entry to indicate if the PYO was approximated or if an exact PYO was provided in the paper. If PYO=POP*TIME then it was approximated (value 1), and if PYO was reported in the paper (even if it is converted to years from days/weeks/months), then it was exact (value 0).

INC. Incidence=(d/PYO)*1,000. Incidence may have been explicitly reported in the paper. However, the likelihoods are derived from *d* and PYO and therefore if incidence was provided, the PYO and *d* were calculated from INC.

INC_NOTES. Description for how incidence values were obtained or derived from information given in the publication.

DIAGNOSTIC. Diagnostic technique used (*Microscopy* or *RDT*). Data based on serology or PCR were not included.

CLINICAL_DEF. Clinical Definition. The definition of a clinical case as given in the paper: fever+any parasitaemia or fever+parasitaemia within a fixed or age-dependent threshold (fixed was used if the study reported both).

CASE_DENS_THRESH. Case definition parasite threshold. Some publications specified a minimum parasite load for a patient to be considered a positive case. If any parasite density was permitted, a threshold of 1 was entered, otherwise the value specified in the paper was entered.

### 4. Incidence population

INC_LAR. Incidence lower age range. If there were multiple age groups studied in one paper, they were entered as separate rows. If no lower age was given, it was assumed to be zero.

INC_UAR. Incidence upper age range. If no upper age was given, it was assumed to be 85.

EIR. Entomological inoculation rate. This was recorded if given in the reference to provide a measure of transmission intensity.

INTERVENTION. Any interventions taking place in the study population. Control and intervention arms should be entered in different rows. For control groups or if there was no intervention, *None* was entered.

### 5. Prevalence data

PR_AUTHOR. The author of the source of the parasite rate (PR) data. If the data was found in the same paper as the incidence data, it was entered as *Same*.

PR_PUBYEAR. The year that the reference that cites the parasite rate was published. If it is the same paper as the incidence paper, it was entered as *Same.*

PR_MONTH. The month prevalence that the survey was conducted. If not provided, *NA* was recorded.

PR_YEAR. The year that the prevalence survey was conducted.

N_SURVEYS. This refers to the number of prevalence surveys (XSS) included in the PR estimate reported. Several studies reported PR values that are averaged from more than one survey.

PR_LAR. Lower age range of individuals tested in XSS. If no lower age was given, it was assumed to be zero.

PR_UAR. Upper age range of individuals tested in XSS. If no upper age was given, it was assumed to be 85.

AGE_MATCH. If the PR age range was the same as the incidence age range, the value was 1. If they did not match, the value was 0. If there was no PR value from the paper, the value was 99.

N. Number of individuals examined in the prevalence survey, or if slide positivity rate was reported, the number of slides examined was used.

N_POS. Number of individuals positive for parasite in question.

N_POS_ADJ. Adjusted number positive based on age-standardized prevalence value (PapPR_Stand or PR_Stand below).

PR. Calculated parasite rate=(N_POS/N).

PR_NOTES. Description of how a concurrent PR estimate was obtained.

PR_DIAGNOSTIC. Diagnostic technique used to identify *P. falciparum* or *P. vivax* prevalence within the population (microscopy or RDT). Data based serology or PCR were not included.

### 6. Matched prevalence data

PfPR2_10. Predicted *Pf*PR values from the *P. falciparum* MAP endemicity surface^7^.

PvPR1_99. Predicted *Pv*PR values from the *P. vivax* MAP endemicity surface^15^.

MAPPR_Stand. Estimate from the *P. falciparum* or *P. vivax* MAP surface age-standardized to the age-range used in the incidence data.

PapPR_Stand. Concurrent PR estimate age-standardized to the incidence age-range.

PR_Stand. If a concurrent PR estimate was available, the age-standardized one is used here, if not, the age-standardized MAP estimate is used.

### 7. Citations

REF_ACD. Reference for the incidence data.

PMID. PubMed identification number for ACD reference. Unpublished sources were left blank, but the type of source (e.g. thesis or conference proceedings) was noted in the full reference given in REF_ACD.

REF_PR. Reference for the PR data (if available).

PMID_PR. PubMed identification for PR reference. Unpublished sources were left blank, but the type of source (e.g. thesis or conference proceedings) was noted in the full reference given in REF_PR. If no concurrently measured PR was found, this was field also left blank.

## Technical Validation

There were 1,680 rows of incidence data following initial data extraction (Data Citation 1). All records were entered by one team member and checked by a second. Cells where there was disagreement were highlighted and checked by a third person where possible. Checking was done to ensure that entries were accurate and that the inclusion criteria outlined above were met. Some exceptions to inclusion criteria described were made to allow for studies used in previous analyses^5,17^ to be added to the database.

To record any exceptions to the inclusion criteria used in the Cameron *et al.*^13^ and Battle *et al.*^14^ studies and to flag other records for potential exclusion in future analyses, an additional field was added to the database (see EXCLUSION field above). The first exclusion, which applied to both *P. falciparum* and *P. vivax* data, was to remove records from different studies that reported the same data (same population at the same time). The records prioritized for inclusion were those that had been included in previous analyses^5,17^. Next, studies that measured both symptomatic and asymptomatic cases that passed the first inclusion stage, but found during validation, were marked for potential exclusion as the incidence measure would not be specific to clinical cases. Studies with unclear methods, such as un-specified frequency of detection or the number of cases or person-time could not be derived, were also marked for potential exclusion. For *P. falciparum*, the remaining potential exclusion criteria based on the analysis by Cameron *et al.*^13^ was to not include studies that (i) had fewer than four age-specific estimates from the same population during the same time to remove studies under-powered for inference of the *P. falciparum* age-incidence relationship and or (ii) where the population were treated presumptively at the start of the transmission season. There were only six *P. falciparum* records excluded from the Cameron *et al.* analysis for presumptive treatment, but it was noted in INTERVENTION field for other records that had been flagged for potential exclusion for other reasons. For the *P. vivax* analysis by Battle *et al.*^14^, studies that made ACD visits more than one month apart were excluded, as were studies conducted prior to 1985. Incidence reports from retrospective analyses or passive case detection (PCD)-only were marked in the EXCLUSION field, as were XSS because they are not longitudinal and measure both symptomatic and asymptomatic cases. The records flagged as PCD and XSS had originally been abstracted because they were used in previous analyses^5,17^.

A summary of the exclusion criteria described above is shown in Table 2 and a schematic of the exclusion procedures is shown in Fig. 1. Table 3 shows the regional distribution of the 328 *P. falciparum* and the 152 *P. vivax* records remaining after the species-specific exclusions described above were applied. The study by Cameron *et al.*^13^ was restricted to Africa, but the lack of data in Africa in the *P. vivax* analysis conducted by Battle *et al.*^14^ represents a genuine absence of *P. vivax* data in the region. Note that all 1,680 records originally abstracted remain in the database so that customized exclusions can be applied for any future analyses using this data.

## Usage Notes

This dataset was generated for the purpose of modelling the relationship between incidence of clinical malaria and the more commonly measured PR. This database has been directly applied to the models described in Cameron *et al.*^13^ and Battle *et al.*^14^, and is similar to the smaller dataset published by Patil *et al.*^5^, with the intention of developing species-specific global burden maps for *P. falciparum* and *P. vivax* malaria. Those in turn directly inform global estimates of the burden of clinical disease attributable to each species. This information is critical to efficiently allocate resources and direct efforts to combat these illnesses.

As described above, this database reports incidence of all infections, not only new infections, and therefore include relapses (*P. vivax* only), recrudescences and reinfections in both the prevalence and incidence measures^27,34,35^. For *P. vivax* in particular, these data could be used in conjunction with data on patients who have received radical cure treatment (with either primaquine or tafenoquine) or treatment without primaquine to determine the incidence of new infections or relapses, respectively. This would be done by taking the overall incidence in a location, as reported here, and subtracting the incidence of new infections from patients treated with a radical cure or without a radical cure. This would be essential data for determining sporozoite- and hypnozoite-specific attack rates, the relative proportions of which may directly inform the character of interventions against endemic malaria.

It has been hypothesised that a key driver of relapse in *P. vivax* is infection with *P. falciparum*. To allow for investigation of the potential interactions between the endemicity of one species on the incidence of another, a prevalence measure for both *P. vivax* and *P. falciparum* is provided for each entry.

This database may also be of use for other analyses of clinical burden. Where possible, data has been disaggregated by age. This allows for studies of how burden of disease changes with age, as was done by Griffin *et al.* using a smaller dataset of *P. falciparum* in children in Africa^12^. The database also contains incidence data from intervention studies with data from both intervention and control arms entered. This would offer insight into the impact of control on incidence of disease as compared to prevalence of infection. Finally, the collection of data from 1985 until the present may improve our understanding in the change of malaria burden over time.

## Additional Information

**How to cite this article:** Battle, K. E. *et al.* Global database of matched *Plasmodium falciparum* and *P. vivax* incidence and prevalence records from 1985–2013. *Sci. Data* 2:150012 doi: 10.1038/sdata.2015.12 (2015).

## Supplementary Material

Supplementary information

## Figures and Tables

**Table 1:** Data records for *P. falciparum* and *P. vivax* by MAP region.

**Region**	***P. falciparum***	***P. vivax***	**Total**
Africa+	661	11	672
America	117	106	223
CSE Asia	409	376	785
Total	1,187	493	1,680

**Table 2:** Exclusion criteria applied to the initial dataset for *P. falciparum* and *P. vivax*.

Potential exclusion criteria*	**Description**	**Pf**	**Pv**	**Total**
Duplicate	Data from different studies reporting the same data or data from the total population where age-specific data were also reported	15	10	25
Asymptomatic	Papers that did not diagnose based on clinical symptoms, but on infection alone, and therefore asymptomatic cases would be included in the incidence estimates	11	6	17
Unclear	Methods regarding the ACD were vague; often the frequency of ACD was not reported	50	36	86
Infrequent ACD	Studies that carried out ACD at intervals greater than 30 days	22	9	31
Pre-1985	Studies that were published after 1985, but contain incidence data gathered before 1985	38	21	59
XSS	Studies that appear to be cross-sectional surveys rather than longitudinal ACD studies	10	10	20
PCD	Studies that appeared to use only passive case detection	54	13	67
<4 age bands	Studies that either did not stratify incidence by age or did so with less than four age groups	859	371	1,230
Approximate person-time	Person-years observed (PYO) was approximated by multiplying the study population by study time period	722	300	1,022
Rx	Population was given presumptive treatment prior to ACD observation period	35	2	37

*Figure 1 illustrates how these criteria were applied to the species-specific data records.

**Table 3:** Data records for used in analysis by Cameron *et al.*^13^ and Battle *et al.*^14^ by MAP region.

**Region**	***P. falciparum*—Cameron** *	***P. vivax*—Battle** †
Africa+	295	—
America	—	43
CSE Asia	—	133
**Total**	**295**	**176**

*The study by Cameron *et al.* was restricted to Africa.

^†^The study by Battle *et al.* was global and therefore lack of records in Africa+ represents a genuine absence of data that matched the inclusion criteria from that region.
